# GMDPtoolbox: A Matlab library for designing spatial management policies. Application to the long-term collective management of an airborne disease

**DOI:** 10.1371/journal.pone.0186014

**Published:** 2017-10-05

**Authors:** Marie-Josée Cros, Jean-Noël Aubertot, Nathalie Peyrard, Régis Sabbadin

**Affiliations:** 1 MIAT - UR 875, INRA, Toulouse, France; 2 AGIR - UMR 1248, INRA/INPT, Toulouse, France; US Department of Agriculture, UNITED STATES

## Abstract

Designing management policies in ecology and agroecology is complex. Several components must be managed together while they strongly interact spatially. Decision choices must be made under uncertainty on the results of the actions and on the system dynamics. Furthermore, the objectives pursued when managing ecological systems or agroecosystems are usually long term objectives, such as biodiversity conservation or sustainable crop production. The framework of Graph-Based Markov Decision Processes (GMDP) is well adapted to the qualitative modeling of such problems of sequential decision under uncertainty. Spatial interactions are easily modeled and integrated control policies (combining several action levers) can be designed through optimization. The provided policies are adaptive, meaning that management actions are decided at each time step (for instance yearly) and the chosen actions depend on the current system state. This framework has already been successfully applied to forest management and invasive species management. However, up to now, no “easy-to-use” implementation of this framework was available. We present GMDPtoolbox, a Matlab toolbox which can be used both for the design of new management policies and for comparing policies by simulation. We provide an illustration of the use of the toolbox on a realistic crop disease management problem: the design of long term management policy of blackleg of canola using an optimal combination of three possible cultural levers. This example shows how GMDPtoolbox can be used as a tool to support expert thinking.

## Introduction

Management problems in ecology and agroecology are complex because several components must be managed together while spatial interactions occur among them. In addition, management actions are applied at a local level while the objective is often defined at a larger level. For instance, for optimizing biodiversity conservation, protection actions may target only a few species or habitats, while the whole biodiversity is of interest. The choice of the target species/habitats depends strongly on the ecological interaction network between the species and habitats [1]. Ecosystem services are usually expected at the regional level, while management actions are applied at the field level [2, 3]. In agroecology, many processes occur at levels higher than the field level because interactions take place among landscape components (commercial fields and interstitial spaces) through biotic and abiotic flows. For instance, erosion problems must be managed collectively at the catchment basin level [4]. In addition, spatial dispersion of pests and beneficials create spatial dependencies between fields and other habitats [5]. A given proportion of refuge fields must be maintained at the landscape level in order to limit adaptation of insects to genetically modified Bt crops [6]. Lastly, management of long-term pesticide durability must be applied at the landscape level for fungicides [7], insecticides [8], and herbicides [9].

Another feature of these management problems is that the objective is a long-term one: biodiversity and production must be preserved in a sustainable way and decisions are not taken once and for all. Instead, sequences of decisions must be taken without a precise and deterministic knowledge of the potentially delayed effects of the decisions on the system [10].

Markov Decision Processes (MDPs [11, 12]) form a suitable framework for modeling and solving problems of sequential decision under uncertainty. A MDP is defined in terms of state variables, action variables, transition probability functions and reward functions. Solving a MDP amounts to finding the policy that optimizes the expected sum of future rewards, over a given time horizon. There exist several freely available toolboxes for solving Markov Decision Processes [13–16]. However, their direct application to domains like ecology or agroecology is difficult when there are a large number of state variables together with a large number of action variables.

Several approaches have been proposed for solving MDPs with multidimensional state and action spaces (FA-FMDPs [17–19]). In general, such methods do not compute an optimal global policy for a given objective, but only an approximate one. A global policy is a set of decision rules that prescribe the actions to apply in any particular entity (e.g. a field, a species) depending on the current state of all the considered entities. In practice, computing and even representing global solution policies for FA-FMDP may quickly become too difficult when the number of state and action variables increases. In addition, it is not always realistic to assume that complete knowledge of the values of all state variables is available when deciding the value of a local management action variable. Therefore, most approaches for solving large FA-FMDPs have tried to overcome this problem by computing approximate policies which are local, in the sense that the decision rule prescribes the action to apply locally, based only on the current states of the few entities in direct interaction.

One such approach is the Graph-based MDP framework (GMDP [20, 21]) and the associate solution algorithms. In a GMDP, each entity is represented as a node of a graph. To each node is associated a pair of state / action variables. The graph edges represent local dependencies in the transition and reward functions. For a fixed policy, the dynamics model is a Dynamic Bayesian Network [22]. Its graphical representation provides an easy interpretation of the local dependencies in the GMDP model. Algorithms dedicated to GMDPs usually find “good” local policies [20, 21], but without any optimality guarantee.

The GMDP framework has already been used to model management problems and to derive policies in various fields: plant disease management [23], human disease management [24], forest management [25], and invasive species control [26].

In this article, we present GMDPtoolbox, a Matlab toolbox which is useful for modeling spatial management problems, for designing and analyzing policies and for comparing given policies by simulation. It provides implementations of the *Approximate Linear Programming* and the *Mean-Field Approximate Policy Iteration* algorithms proposed in [20]. We first briefly describe the GMDP framework, as well as the two above-mentioned algorithms. Then, we describe the functionalities of the toolbox: GMDP solution functions, policy analysis tools and documentation. Finally we provide an illustration of the use of the toolbox, on a realistic crop disease management problem: the design of optimal long term management policies (or strategies) of blackleg on canola (UK: Phoma stem canker on oilseed rape), through the use of three management levers: specific genetic resistance, tillage and cultural control. We show how spatial interactions can be modeled and how collective (at the scale of an agricultural area) and integrated (combining several action levers) control policies can be proposed to support expert thinking.

## The GMDP framework

As a tutorial example, we describe the GMDP framework with the particular interpretation of entities as sites of a spatial area. A site can be a crop field, a forest stand, etc. However, interactions in a GDMP are not limited to the modeling of spatial interactions. They can be, for instance, trophic or ecological interactions.

### Definitions

A discrete-time GMDP is defined by a 5-tuple of variables <*S*, *A*, *N*, *p*, *r*> (see Table 1 for a list of variables definition) where:

- *S* is the state space,
  *S* = *S*_1_ × … × *S*_*n*_ with *S*_*i*_ the finite state space of site *i*.
- *A* is the action space,
  *A* = *A*_1_ × … × *A*_*n*_ with *A*_*i*_ the finite action space of site *i*.
- *N* is the set of sites neighbors set,
  *N* = {*N*_*i*_, ∀*i* = 1, …, *n*} where *N*_*i*_ ⊆ {1, …, *n*} is the set of neighbors of site *i*. Note that it is possible that *i* ∈ *N*_*i*_, but this is not mandatory.
- *p* is the set of local sites transition probability functions,
  $p=\{p_{i}\left(s_{i}^{\prime }|s_{N_{i}},a_{i}\right),\;\forall i=1,\cdots ,n,\;\forall s_{i}^{\prime },s_{N_{i}},a_{i}\}$, where $p_{i}\left(s_{i}^{\prime }|s_{N_{i}},a_{i}\right)$ is the (stationary) probability for site *i* of transitioning to $s_{i}^{\prime }$ at time *t* + 1 given that at time *t* the neighborhood of the site is in state *s*_*N*_*i*__ = {*s*_*j*_, *j* ∈ *N*_*i*_} and action *a*_*i*_ is performed.
  The global transition probability is factored according to the local transition probabilities: if $s=\left(s_{1}\ldots s_{n}\right),s^{\prime }=\left(s_{1}^{\prime }\ldots s_{n}^{\prime }\right)$ and *a* = (*a*_1_…*a*_*n*_) are global state and action vectors,
  $$\begin{array}{c} p\left(s^{\prime }|s,a\right)=\prod_{i=1}^{n}p_{i}\left(s_{i}^{\prime }|s_{N_{i}},a_{i}\right),\forall s\in S,\forall s^{\prime }\in S,a\in A \end{array}$$
- *r* is the set of local sites reward functions
  *r* = {*r*_*i*_(*s*_*N*_*i*__, *a*_*i*_), ∀*i* = 1, …, *n*, ∀*s*_*N*_*i*__, ∀*a*_*i*_}
  with *r*_*i*_ the reward obtained from site *i* at time *t* when the neighborhood of site *i* is in state *s*_*N*_*i*__ and action *a*_*i*_ is performed.
  The global reward is the sum of the local ones:
  $$\begin{array}{c} r\left(s,a\right)=\sum_{i=1}^{n}r_{i}\left(s_{N_{i}},a_{i}\right),\forall s\in S,\forall a\in A. \end{array}$$

**Table 1:** Variables related to GMDP framework definition.

Variable	Definition
*n*	number of sites
*i*	a site number
*N*	set of sites neighbors set, *N* = {*N*_*i*_, ∀*i* = 1,…, *n*}
*N*_*i*_	set of neighbors of site *i*, *N*_*i*_ ⊆ {1, …, *n*}
*S*	state space, *S* = *S*_1_ × … × *S*_*n*_
*S*_*i*_	finite state space of site *i*
*s*, *s*′	state of all sites, *s* ∈ *S*, *s*′ ∈ *S*, *s* = {*s*_1_, …, *s*_*n*_}, $s_{i}^{\prime }=\left\{s_{1}^{\prime },\cdots ,s_{n}^{\prime }\right\}$
$s_{i},s_{i}^{\prime }$	state of site *i*, $s_{i}\in S_{i},s_{i}^{\prime }\in S_{i}$
*s*_*N*_*i*__	state of neighbors of field *i*, *s*_*N*_*i*__ = {*s*_*j*_, *j* ∈ *N*_*i*_}
*s*^*t*^	state of sites at time *t*, *s*^*t*^ ∈ *S*
*A*	action space, *A* = *A*_1_ × … × *A*_*n*_
*A*_*i*_	finite action space of site *i*
*a*	action performed on all fields, *a* ∈ *A*
*a*_*i*_	action performed on field *i*, *a*_*i*_ ∈ *A*_*i*_
*p*	set of stationary local sites transition probability functions
$p_{i}\left(s_{i}^{\prime }|s_{N_{i}},a_{i}\right)$	probability for site *i* of transitioning to $s_{i}^{\prime }$ at time *t*+1 given that at time *t* the neighborhood of the site is in state *s*_*N*_*i*__ and action *a*_*i*_ is performed at time *t*
*r*	set of stationary local sites reward functions
*r*_*i*_(*s*_*N*_*i*__, *a*_*i*_)	reward obtained from site *i* at time *t* when the neighborhood of site *i* is in state *s*_*N*_*i*__ and action *a*_*i*_ is performed
*δ*	stationary decision rule or policy, a function *δ*: *S* → *A* assigning an action to every state
*v*_*δ*_(*s*)	infinite horizon discounted value of a policy *δ* applied with initial state *s*
*γ*	discount factor

In a usual MDP [11], a function *δ*: *S* → *A* assigning an action to each state is called a *stationary decision rule* or *policy*. Once a policy *δ* is fixed, the MDP defines a stationary Markov Chain over *S*, with transitions *p*_*δ*_(*s*′|*s*) = *p*(*s*′|*s*, *δ*(*s*)). The infinite horizon discounted value *v*_*δ*_(*s*) of a policy *δ*, applied to a MDP with initial state *s*, is defined as:

$$\begin{array}{c} v_{\delta }\left(s\right)=E\left[\sum_{t=0}^{+\infty }\gamma^{t}r\left(s^{t},\delta \left(s^{t}\right)\right)|s^{0}=s\right],\forall s\in S. \end{array}$$

The expectation is taken over all possible trajectories 〈*s*^0^, *δ*(*s*^0^), *s*^1^, …, *s*^*t*^, *δ*(*s*^*t*^), … 〉 starting from the initial state *s*^0^ and applying policy *δ*. The discount factor, 0 ≤ *γ* < 1, ensures that the above infinite sum converges. It also takes into account the fact that there is a difference between the “future value” of a reward and the “present value” of the same reward. The problem of finding the optimal policy for a stationary MDP, or solving the MDP, can be written as:

$$\begin{array}{c} \text{Find}\;\delta^{*},S\to A,\;\text{s.t.}\;v_{\delta^{*}}\left(s\right)\geq v_{\delta }\left(s\right),\forall s\in S,\forall \delta \;\left(S\to A\right)\;. \end{array}$$

It has been shown that there always exists an optimal policy [11], and that it can be computed in time polynomial in the size of *S* and *A*, using *Stochastic Dynamic Programming* algorithms such as *Policy Iteration* and *Value Iteration*, or *Linear Programming* algorithms [11].

Since a GMDP is a particular case of MDP, it can be solved using MDP solution algorithms. However, the complexity of these algorithms, which is polynomial in |*S*| and |*A*|, is exponential in *n*. Thus, they are impractical when *n* becomes large. Furthermore, a MDP solution policy, *δ*: *S* → *A* also takes exponential space to represent.

For all these reasons, only approximate solution policies are usually looked for in GMDP problems: the search space is limited to a subset of policies that exploit the notion of neighborhood, namely the set of *local policies*. A policy *δ*: *S* → *A* is said to be *local* if and only if *δ* = (*δ*_1_, …, *δ*_*n*_) where *δ*_*i*_: *S*_*N*_*i*__ → *A*_*i*_ (instead of *δ*_*i*_: *S* → *A*_*i*_). It means that the choice of the action applied on site *i* depends only on the state of its neighbor sites (instead of the state of all sites).

### Two algorithms for approximate resolution of GMDP

The two algorithms implemented in GMDPtoolbox provide local policies by approximating the optimal solution of a GMDP [20]. The first one, referred to as MF-API, exploits the structure of the neighborhood relationships of the GMDP and computes a *mean-field approximation* of the value function of a policy. This algorithm belongs to the family of *Approximate Policy Iteration* (API) algorithms [27]. The second one is a specific *Approximate Linear Programming* algorithm derived from the general class of ALP algorithms [28] and adapted to the GMDP framework. Previous experimental comparisons have shown that the two algorithms provide local policies of similar quality, outperforming naive policies such as greedy or random policies. However, the MF-API algorithm provides a higher-quality approximation of the expected value of the returned policy than the ALP algorithm, which is faster. Thus, the two methods can be seen as complementary. We refer the reader to [20] for a full description of these two algorithms and their comparison.

## GMDPtoolbox

This section describes i) how the Matlab GMDPtoolbox can be used to model spatial management problems; ii) how the value of a policy is computed; and finally iii) how to generate spatio-temporal simulations of the system under the application of a given policy for given initial states. These aspects are illustrated on a generic toy problem in the domain of crop protection.

**Description of a simple epidemiological toy model.** For didactic purposes, we consider a simple implementation of a generic epidemiological toy model with GMDPtoolbox. We consider a situation with 3 commercial fields in which 2 different crops can be sown. One of these crops induces an important profit, however, it is susceptible to a pathogen, and when infected, the profit is reduced. A second crop can be sown, instead, which induces a lower profit. This second crop is not susceptible to the pathogen and induces its elimination from the field (this is the main interest of this crop). The problem is then to decide a long-term crop policy (or strategy), at the scale of the landscape (three fields).

**Fig 1:**
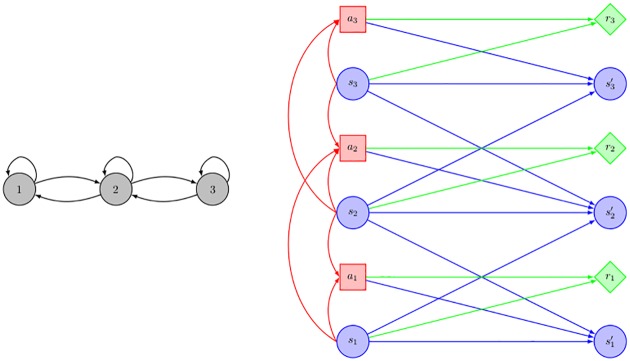
Toy epidemiological management problem: (A) graphical representation of the neighborhood relationships and (B) GMDP transition, reward and policy structures. Blue (respectively red, green) nodes represent state variables (respectively action variables, reward functions). Blue (respectively red, green) arrows model the influence on states variables (respectively actions variables, rewards functions).

Each field can be described by two states: uninfected (coded 1) or infected (coded 2), |*S*_*i*_| = 2,∀*i* = 1, 2, 3. Crop management decisions are taken with a yearly time step and only two actions can be applied to each field: either the high-profit susceptible crop is sown (coded 1) or the low-profit resistant one (coded 2), |*A*_*i*_| = 2,∀*i* = 1, 2, 3. The problem is to identify the policy that maximizes the expected cumulative profit on a long-term basis. The topology of the considered area can be represented by a graph (see Fig 1A). In this graph, each node represents a commercial field. A directed edge between two nodes represents potential contamination flows. The neighborhood relationships are here symmetric: *N*_1_ = {1, 2}, *N*_2_ = {1, 2, 3}, *N*_3_ = {2, 3}. The GMDP representation of transitions, policies and rewards structures is displayed in Fig 1B.

Transition probabilities are defined from the following probabilities:

1. the probability *p*_*ϵ*_ of long-distance contamination of fields containing susceptible crops,
2. the probability *p*_*c*_ that a field containing a susceptible crop be contaminated from an infected neighboring field.

The probability that a non-infected field at time step *t* with *m*_*i*_ infected neighboring fields moves to state infected at time *t* + 1 is then defined by:

$$\begin{array}{c} p_{\epsilon }+\left(1-p_{\epsilon }\right)\left(1-{\left(1-p_{c}\right)}^{m_{i}}\right). \end{array}$$

Note that a field can be non-infected either because the non-susceptible crop was used or because the susceptible one was, but did not get infected by the pathogen. In addition, if a field was infected at time *t* and is still sown with the susceptible crop (*a*_*i*_ = 1), then the field remains infected at time *t* + 1 with probability 1. If a non-susceptible crop is used (*a*_*i*_ = 2) at time *t* then the field becomes uninfected with probability 1 at *t* + 1. Profit for each field is impacted by the nature of the chosen crop (*a*_*i*_ = 1, 2), and its state (infected or uninfected). The minimum profit (noted *r*_0_) is obtained when the non-susceptible crop is used (*a*_*i*_ = 2), while the maximum profit (*r*_*m*_ + *r*_0_) is obtained when the susceptible crop is sown and the field is not infected. An intermediate profit (*r*_*m*_/2 + *r*_0_) is obtained when the susceptible crop is used while the field is infected. For a field *i* in state *s*_*i*_ at time *t*, the rewards obtained when action *a*_*i*_ is performed are given in Table 2. In order to simplify the analysis of optimal policies, we arbitrarily fixed *r*_0_ = 0 in the Toolbox example implementation.

**Table 2:** Reward when field *i* is in state *s*_*i*_ at time *t* and action *a*_*i*_ is performed.

	*a*_*i*_ = 1	*a*_*i*_ = 2
*s*_*i*_ = 1	*r*_*m*_ + *r*_0_	*r*_0_
*s*_*i*_ = 2	*r*_*m*_/2 + *r*_0_	*r*_0_

**Fig 2:**
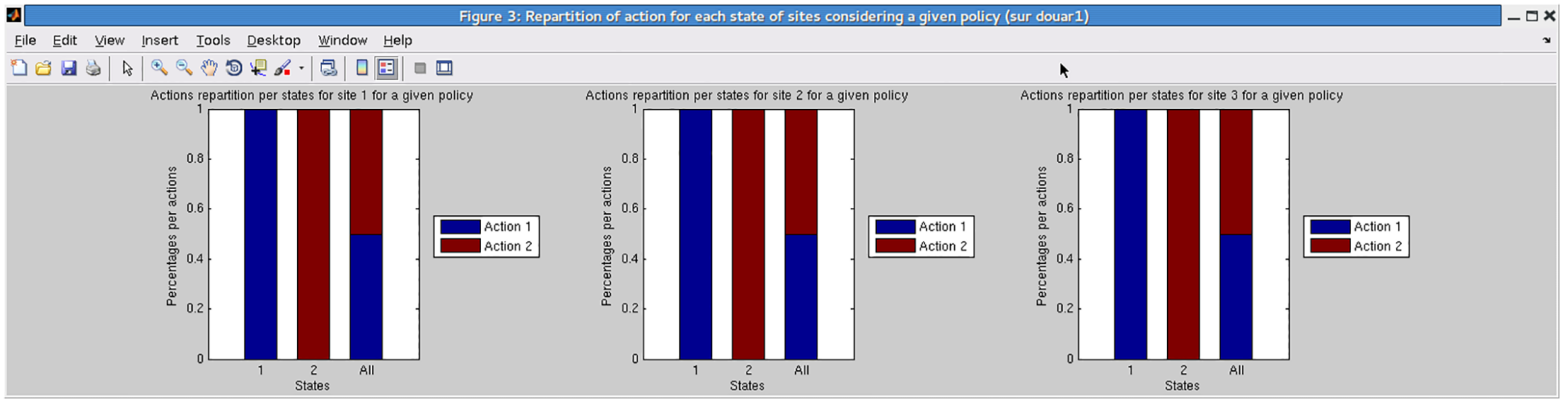
Toy epidemiological management problem: proportions of neighborhood configurations for which the GMDP policy prescribes action 1 and action 2, at each of the three sites.

**Describing and analyzing a policy.** On this toy example the MF-API and the ALP solution algorithms lead to the same policy (experiments were run with a discount factor equal to 0.95). This policy can be difficult to interpret since it corresponds to a set of local functions *δ*_*i*_ from *S*_*N*_*i*__ to *A*_*i*_. In GMDPtoolbox, one of the proposed visualizations enables to show the proportion of each action applied for each site, depending on the site state (see Fig 2). From these graphics we can see that in this very simple example the policy amounts to the following simple rules, that depend only on the site status and not on the status of the neighboring fields: if field *i* is uninfected (site state 1) then use the high-profit susceptible crop (action 1); if field *i* is infected (site state 2) then use the low-profit resistant one (action 2).

**Fig 3:**
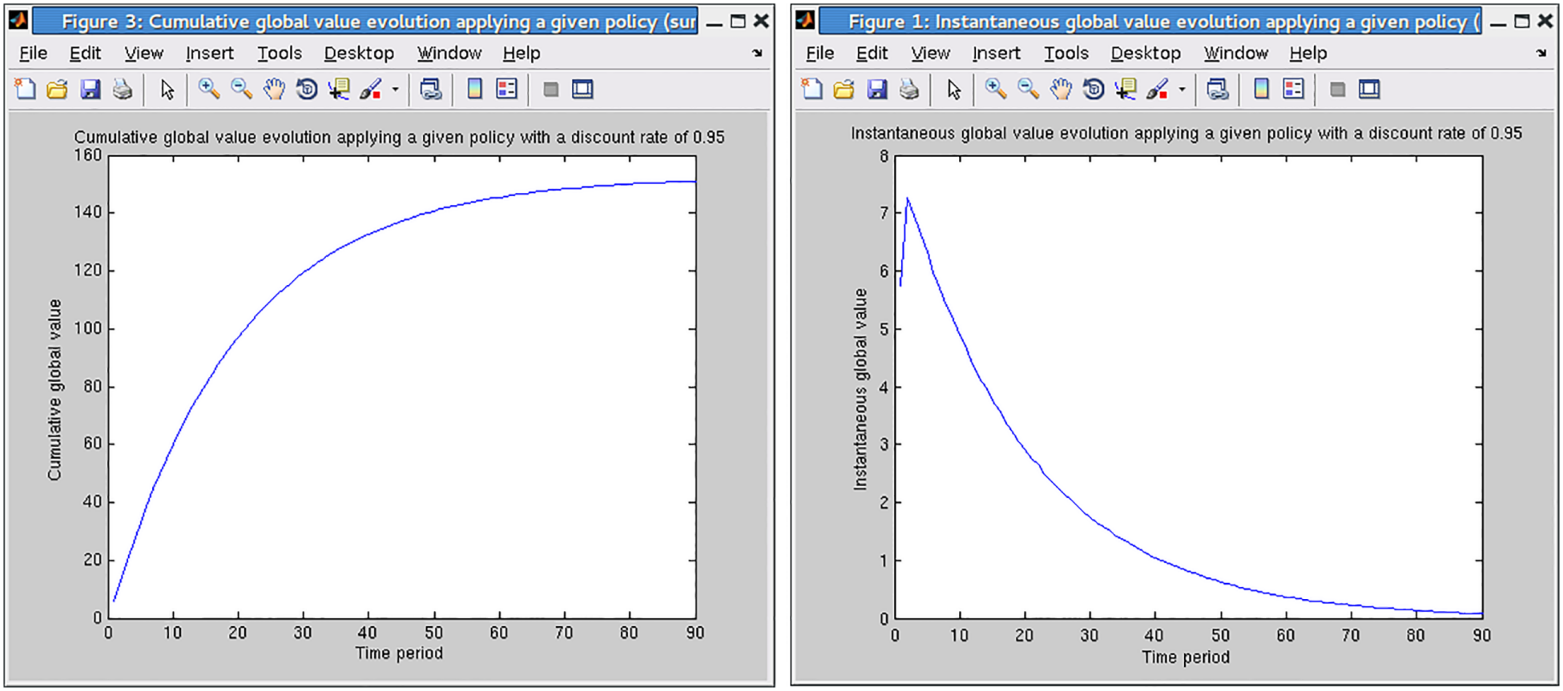
Toy epidemics management problem: (A) cumulative and (B) instantaneous discounted global reward of the GMDP policy, estimated by Monte Carlo simulation.

**Simulating the effect of a policy.** In GMDPtoolbox, the evolution of the cumulative global value of the GMDP policy (*i.e.* the truncated infinite horizon discounted value) can be obtained by Monte Carlo approximation, using simulations (Fig 3A). The GMDPtoolbox also provides a graphical representation of the instantaneous global value, *i.e.* the expectation of the (discounted) sum of rewards at a given time step, over all sites (Fig 3B).

Other functions enable quantification of the contribution of each site to the cumulative global value, and the time spent by each site in the different possible states.

**General information.** GMDPtoolbox relies on the free toolbox graphViz4Matlab for three functions that display graphs. One of the solution functions relies on the Matlab Optimization toolbox. Furthermore the two solution functions can be accelerated by using the Matlab Parallel Computing toolbox. A complete description of GMDPtoolbox is available at http://www.inra.fr/mia/T/GMDPtoolbox and the source code is available from Matlab Central (https://fr.mathworks.com/matlabcentral/fileexchange/49101-graph-based-markov-decision-processes-gmdp-toolbox) or the project Forge (https://mulcyber.toulouse.inra.fr/projects/gmdptoolbox).

## Application of the GMDP framework to the long-term collective management of an airborne disease at the landscape level

### Description of the problem

There is a need to limit the structural dependency of European agriculture on pesticides while, at the same time, to maintain satisfactory levels of production and income for farmers. The use of resistant cultivars is the cornerstone of Agroecological Protection against plant pathogens. However, these resistances can be overcome within a few years [29]. There is therefore a need for tools that help design collective management policies which do not rely only on resistant cultivar and mobilize several management levers instead.

In order to illustrate the interest of the GMDP framework and GMDPtoolbox in this context, we consider a simplified management problem that focuses on the long-term collective management of an important disease worldwide: blackleg on canola, caused by the *Leptosphaeria maculans / biglobosa* complex species [30]. Epidemics of blackleg on canola are initiated by infected stubble, remaining on the soil surface after harvest of canola, and that produces ascospores after a period of maturation. These spores are wind-dispersed and can produce leaf spots on seedlings and young canola plants in proper conditions of infection [30]. Once the fungus has infected a leaf, it systematically colonizes the plant and produces a canker, located at the basal stem and the crown, that develops after winter. Control of blackleg on canola mainly relies on the use of cultivar with specific and/or quantitative resistances and cultural controls. Whenever possible, soil tillage should be adopted to reduce the quantity of available primary inoculum [31]. Because of spore dispersal, trying to contain the disease at the field level only is not sufficient. Collective policies at a regional level should be more efficient and more sustainable.

We designed a qualitative model that represents the impact of cropping practices on epidemics of blackleg on canola and the changes over time of a *Leptosphaeria maculans* population. A three-year rotation is assumed for the entire region: fields are successively sown with canola, then wheat, then barley. This is a typical rotation in France. Primary inoculum is produced in wheat fields from infected stubble left on the soil surface after the harvest of canola. Then spores reach neighbor canola fields by dispersion. The genetic structure of the pathogen population is described in terms of proportion of virulent pathotypes to the considered specific resistance. Then the qualitative model of the pathogen spatio-temporal dynamics corresponds to a downgrading of the SIPPOM-WOSR model [32]. This model describes the effects of cropping systems and their spatial arrangement at the landscape level, along with the effects of weather on the genetic structure of *L. maculans* populations, epidemics, and yield losses on canola.

We considered 3 management levers (*action variables* in the GMDP framework): cultivar choice (2 choices: with of without a specific resistance), canola management plan (2 choices: favorable or unfavorable to blackleg; these cultural modes differ in terms of soil nitrogen content, sowing date and sowing density [32, 33]) and tillage (2 choices: plowing or not after the harvest of canola). Each canola field can thus be managed with 2^3^ = 8 possible options. These actions are applied to canola fields on a yearly basis. The wheat and barley fields are assumed to be managed to provide the same annual harvest over years. Using a simple damage function, yield losses were estimated and the economic performances of cropping practices were calculated as a function of economic drivers (*i.e.* crop management cost and canola prices).

The GMDPtoolbox is used to test the effect of 3 contrasted policies corresponding to 3 different attitudes to control blackleg. These policies are compared to the policy computed by the ALP algorithm (referred to as the GMDP policy). All these policies adapt the action choice on each field, on a yearly basis, as a function of the neighboring field states or indicators calculated at the regional scale. The policies are evaluated with regard to the cumulative global gross margin of farmers in the considered region, on a long term basis.

### The GMDP model

The variables of the model are listed in Table 3.

**Table 3:** Variables related to the GMDP model of long-term collective management of an airborne disease at the landscape level.

Variable	Definition
*I*_*i*_	level of inoculum on infected stubble in field *i*, to be interpreted as a low, medium or high number of ascospores per soil surface unit, associated to a low, medium or high percentage of cankered area in the stem section at the crown level ([0 25[, [25 50[ and [50 100]; respectively encoded 1, 2, 3).
*V*_*i*_	level of percentage of virulent spores on infected stubble in field *i*, to be interpreted as a low, medium or high percentage ([0 5[, [5 50[ and [50 100]; respectively encoded 1, 2, 3).
*CC*_*i*_	cultivar choice in canola field *i*: resistant or susceptible (encoded 1, 2)
*CM*_*i*_	crop management in canola field *i*: cautious practice enabling to decrease the risk of infection (early sowing date, low soil nitrogen content and low sowing density) or standard one (with a higher infection risk) (encoded 1, 2).
*W*_*i*_	plowing (tillage operations before canola sowing include plowing) or not in canola field *i* (encoded 1, 2).
*NW*_*i*_	set of neighbor wheat fields of field *i* sown with wheat.

**State of field** *i*. The state *s*_*i*_ of field *i* is either canola (c), barley (b) or represented by a pair (*I*_*i*_, *V*_*i*_) if the field is sown with wheat (which will contain canola stubbles from previous year), with

- *I*_*i*_ ∈ {1, 2, 3} is the level of inoculum on infected stubble in field *i*, to be interpreted as low, medium or high.
- *V*_*i*_ ∈ {1, 2, 3} is the level of percentage of virulent spores on infected stubble in field *i*, to be interpreted as low, medium or high.

The correspondence between global states numbering and the values taken by *I*_*i*_ and *V*_*i*_ variables is given in Table 4.

**Table 4:** Encoding of the 9 possible states when field *i* is a wheat field.

*s*_*i*_	*I*_*i*_	*V*_*i*_
1	1	1
2	1	2
3	1	3
4	2	1
5	2	2
6	2	3
7	3	1
8	3	2
9	3	3

**Action in field** *i*. For fields in wheat or barley, no specific action is applied. For a field *i* in canola, the action *a*_*i*_ is a triple (*CC*_*i*_, *CM*_*i*_, *W*_*i*_) corresponding to

- *CC*_*i*_ (cultivar choice) equal to resistant (encoded by 1) or susceptible (encoded by 2).
- *CM*_*i*_ (crop management). Two crop management plans are considered: practice 1 (cautious) enables to decrease the risk of infection (with an early sowing date, a low soil nitrogen content and a low sowing density) while practice 2 (standard crop management) has a higher infection risk.
- *W*_*i*_ (plowing) equal to 1 if tillage operations before canola sowing include plowing and 2 otherwise.

When the considered field is a wheat field, 9 states are possible (3 possible levels of primary inoculum production x 3 proportions of virulent spores against a given specific resistance). Thus, in total, each state variable *s*_*i*_ has 11 possible values. When a field state variable is in state “canola”, there are 8 possible action variable values (2 possible tillage operations times 2 possible cultivar choices times 2 crop managements), while there is only one available action value when the field is in the 10 other possible states. The correspondence between actions numbering and the values taken by *CC*_*i*_, *CM*_*i*_ and *W*_*i*_ variables is given in Table 5.

**Table 5:** Encoding of the 8 possible actions when field *i* is a canola field.

*a*_*i*_	*CC*_*i*_	*CM*_*i*_	*W*_*i*_
1	1	1	1
2	1	1	2
3	1	2	1
4	1	2	2
5	2	1	1
6	2	1	2
7	2	2	1
8	2	2	2

**Neighborhood relations, *N*.** The fields are modeled as the cells of a regular grid. We define the neighbors of field *i* as the four closest fields (north, south, east, west). This results from the assumption that the landscape experiences only four wind directions (north-south, south-north, east-west, and west-east).

**Fig 4:**
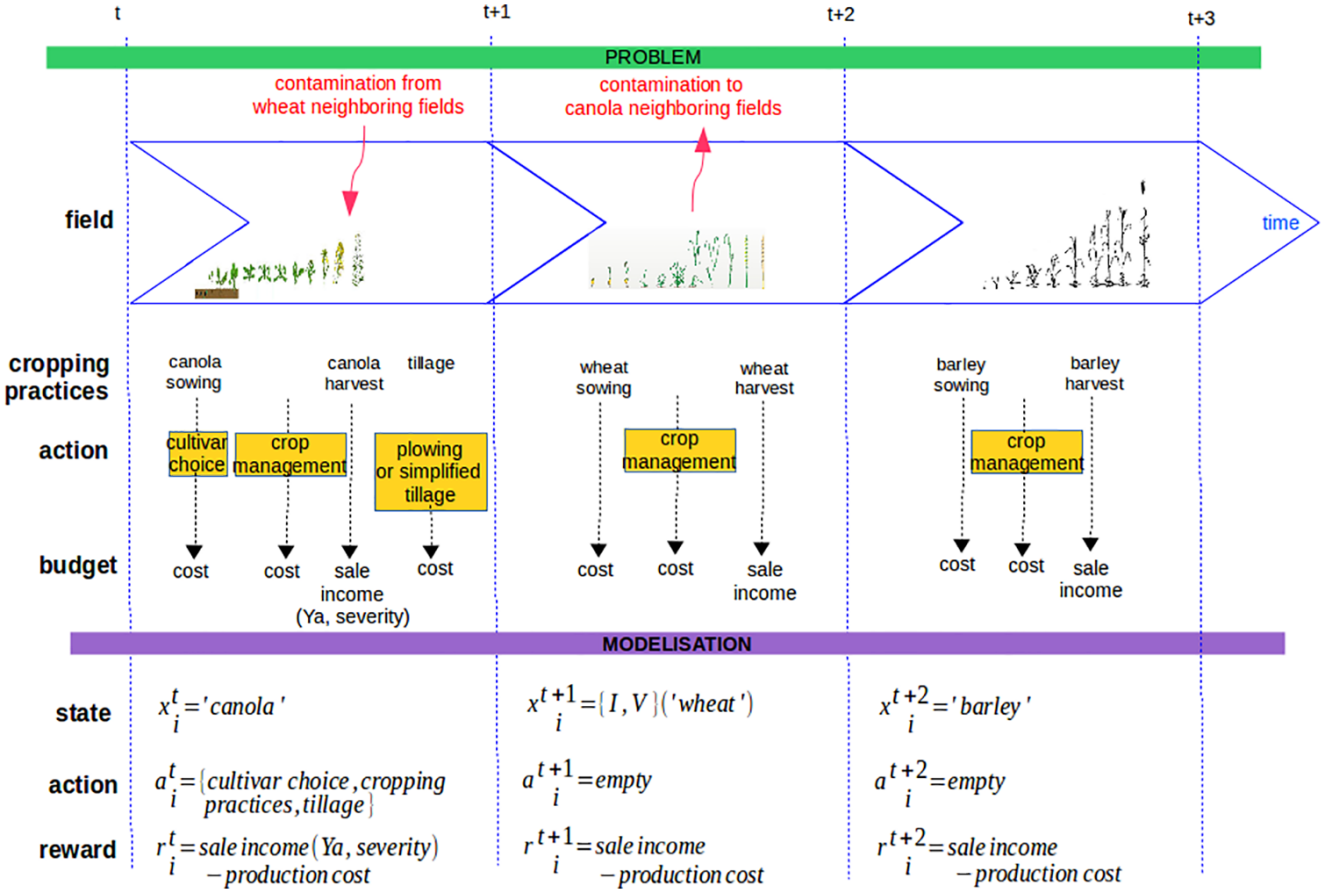
Representation of the succession of events that occur on a field within one year for the three considered crops.

**Transition probability function, *p*.** The succession of events that occur in a field during a cropping season is represented graphically on Fig 4. When field *i* in year *t* is a wheat field, it will be a barley field the next year, while when field *i* at year *t* is a barley field, it will be a canola field the next year (both transition are deterministic). When field *i* at year *t* is a canola field, it will be a wheat field the next year, and the transition to pairs $\left(I_{i}^{t+1},V_{i}^{t+1}\right)$ is stochastic and depends on the state of the neighboring fields of *i* which are in wheat at year *t*, and on action $a_{i}^{t}$. In this case, $P(s_{i}^{t+1}|s_{N_{i}}^{t},a_{i}^{t})$ becomes

$$\begin{array}{ccc} & & P(s_{i}^{t+1}|s_{N_{i}}^{t},a_{i}^{t})\\ & & =P(V_{i}^{t+1},I_{i}^{t+1}|V_{NW_{i}}^{t},I_{NW_{i}}^{t},CC_{i}^{t},W_{i}^{t},CM_{i}^{t})\\ & & =P\left(V_{i}^{t+1}|V_{NW_{i}}^{t},I_{NW_{i}}^{t},CC_{i}^{t}\right)\times P\left(I_{i}^{t+1}|V_{NW_{i}}^{t},I_{NW_{i}}^{t},CC_{i}^{t},W_{i}^{t},CM_{i}^{t}\right) \end{array}$$

where *NW*_*i*_ is the set of indices of the neighboring fields of *i* sown with wheat when *i* is a canola field. The complete definition of the transition probability function is given in the Supporting Information S1 Appendix.

**Rewards.** The yearly reward at the field level is defined as the gross margin: the difference between the income from crop production selling, and the production costs. Its expression depends on the cultivated crop (the parameters of the reward function are described in Table 6): If at time *t* field *i* is a wheat field

$$\begin{array}{c} r\left(s_{N_{i}}^{t},a_{i}^{t}\right)=\beta_{i}\left(SI_{w}-C_{w}\right). \end{array}$$

If at time *t* field *i* is a barley field

$$\begin{array}{c} r\left(s_{N_{i}}^{t},a_{i}^{t}\right)=\beta_{i}\left(SI_{b}-C_{b}\right). \end{array}$$

If at time *t* field *i* is a canola field

$$\begin{array}{c} r\left(s_{N_{i}}^{t},a_{i}^{t}\right)=\sum_{s_{i}^{t+1}}\rho \left(s_{i}^{t},s_{i}^{t+1},a_{i}^{t}\right)p_{i}\left(s_{i}^{t+1}|s_{N_{i}}^{t},a_{i}^{t}\right). \end{array}$$

with

$$\begin{array}{c} \rho \left(s_{i}^{t},s_{i}^{t+1},a_{i}^{t}\right)=\beta_{i}[Y\left(CC_{i}^{t},CM_{i}^{t}\right)\times (1-RYL\left(I_{i}^{t+1}\right)\times \pi_{c}-C_{CM}\left(CM_{i}^{t}\right)-C_{W}\left(W_{i}^{t}\right). \end{array}$$

**Table 6:** Parameters defining the reward function in the GMDP model of management of blackleg on canola.

Parameter	Definition	Unit	Value	Source
*β*_*i*_	surface area of field *i*	ha	25	[34]
*SI*_*b*_	income from barley fields	€.ha^-1^	1040.20	http://faostat3.fao.org
*C*_*b*_	barley production cost	€.ha^-1^	654	[35]
*SI*_*w*_	income from wheat fields	€.ha^-1^	1184.27	http://faostat3.fao.org
*C*_*w*_	wheat production cost	€.ha^-1^	714	[35]
*Y*(1, 1)	attainable canola yield with resistant cultivar and cautious canola management plan	kg.ha^-1^	3000	hypothetical value derived from http://faostat3.fao.org
*Y*(1, 2)	attainable canola yield with resistant cultivar and standard canola management plan	kg.ha^-1^	3500	hypothetical value derived from http://faostat3.fao.org
*Y*(2, 1)	attainable canola yield with sensitive cultivar and cautious canola management plan	kg.ha^-1^	3500	hypothetical value derived from http://faostat3.fao.org
*Y*(2, 2)	attainable canola yield with sensitive cultivar and standard canola management plan	kg.ha^-1^	4000	hypothetical value derived from http://faostat3.fao.org
*RYL*(1)	canola relative yield loss for low injury (level 1)	-	0.0133	[32]
*RYL*(2)	canola relative yield loss for medium injury (level 2)	-	0.0735	[32]
*RYL*(3)	canola relative yield loss for high injury (level 3)	-	0.3283	[32]
*π*_*c*_	canola selling price	€.kg^-1^	0.35	http://faostat3.fao.org
*C*_*CM*_(1)	cost of cautious canola management plan	€.ha^-1^	637	[35, 36]
*C*_*CM*_(2)	cost of standard canola management plan	€.ha^-1^	681	[35, 36]
*C*_*W*_(1)	additional cost of plowing	€.ha^-1^	64	[35]
*C*_*W*_(2)	additional cost without plowing	€.ha^-1^	0	[35]

We considered 500 m x 500 m square fields. This choice is justified by the fact that canola fields further than 500 m away from primary inoculum sources are considered safe with regards to major *Lepstophaeria maculans* infections [34]. With this size of fields, only neighboring fields with infected stubble at soil surface can infect a given canola field. Income from wheat and barley fields (2008-2012 French average yield times 2008-2012 average selling price), as well as canola selling price, were taken from a FAO database (http://faostat3.fao.org). Production costs for wheat and barley fields were estimated by adding operating costs and mechanization labor costs [35]. Attainable canola yields with different crop management plans and cultivar susceptibilities were hypothesized. The three relative yield losses were calculated using the damage function proposed by [32] assuming a Disease Index of 1, 3, and 7 for low, medium and high inoculum levels respectively. Costs of canola management plans were estimated by associating cropping operations [36] with their costs [35] and adding them up (see Table 6).

### The three contrasted policies and the GMDP policy

We consider 3 contrasted policies that correspond to very distinct crop management policies for canola. The first one, the *cultural control* policy, never uses the resistant cultivar and always applies the canola management plan which is the least favorable to blackleg together with plowing: *CC* = 2, *CM* = 1, *W* = 1. On the contrary, the *systematic* policy relies on a permanent use of the resistant cultivar, without plowing and with standard canola management plan: *CC* = 1, *CM* = 2, *W* = 2. Finally, the *integrated* policy is adaptive: if a canola field has no neighbor fields in wheat then the chosen cultivar is sensitive, associated to a standard canola management plan and simplified tillage (action *CC* = 2, *CM* = 2, *W* = 2). Otherwise, if either the maximal level of inoculum or the maximal percentage of virulent spores among the neighbor fields in wheat is in the highest state (*i.e.* 3), cautious decisions are applied: use of resistant cultivar associated to cautious canola management plan and plowing after harvest of canola (*CC* = 1, *CM* = 1, *W* = 1). In all other situations, the sensitive cultivar is used, in association with the canola management plan least favorable to blackleg together with plowing (action *CC* = 2, *CM* = 1, *W* = 1).

It is not straightforward to interpret the optimized GMDP policy from its expression as a function, since the sum over fields of the possible states of the neighborhood is equal to 980,000. We observed that the advocated action (when the field is sown with canola) is to choose sensitive cultivar, standard canola management plan and simplified tillage (action *CC* = 2, *CM* = 2, *W* = 2) for 88% of the cases and to add plowing (action *CC* = 2, *CM* = 2, *W* = 1) for the other 12%. To understand what are the characteristics of the neighbor states that lead to one choice or the other, a CART (Classification And Regression Tree) model was used (Matlab function fitctree). We obtained that the GMDP policy was very well summarized by the following rule (precision of the CART model was of 97%).

**IF** (i) the average of the level of inoculum is low (${\bar{I}}_{NW_{i}}^{t}=1$) and the average of the level of virulent spores is not high (${\bar{V}}_{NW_{i}}^{t}<3$) or (ii) the average of the level of inoculum is low (${\bar{I}}_{NW_{i}}^{t}=1$) and the average of virulent spores is high (${\bar{V}}_{NW_{i}}^{t}=3$) and less than 4 neighbor fields are in wheat or (iii) the average of the level of inoculum is medium (${\bar{I}}_{NW_{i}}^{t}=2$) and the average of virulent spores is medium (${\bar{V}}_{NW_{i}}^{t}=2$) and only one neighbor field is in wheat

**THEN** cultivar is sensitive, associated to a standard canola management plan and simplified tillage (action *CC* = 2, *CM* = 2, *W* = 2)

**ELSE** add plowing (action *CC* = 2, *CM* = 2, *W* = 1).

It can be noted that the long-term optimized GMDP policy does not make use of the cultivar resistance but relies on plowing.

### Comparison of policies’ long term efficiency

**Fig 5:**
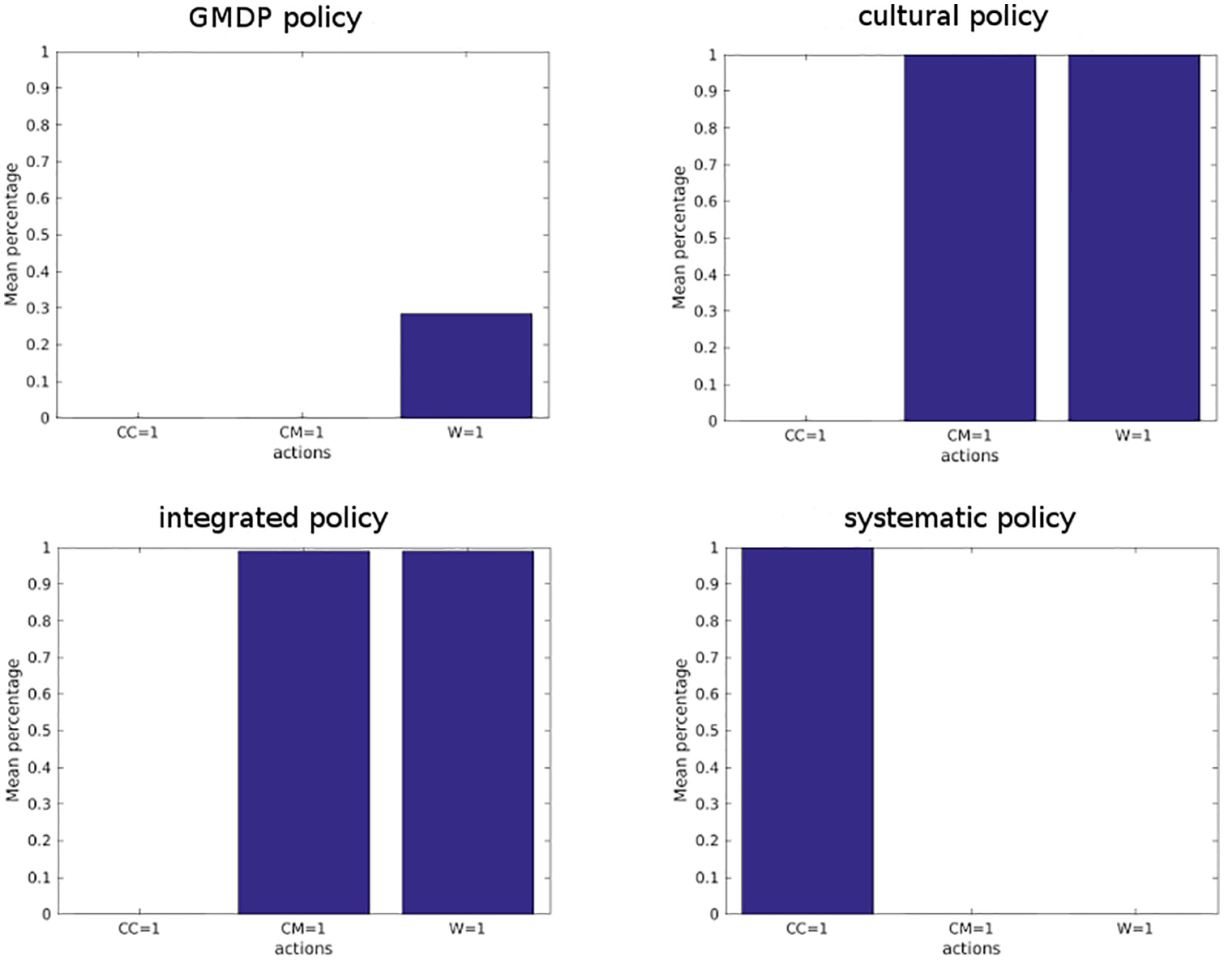
Proportion of use of each action modality, in average over 100 simulations of 100 years of blackleg dynamics. A bar indicates the proportion of times modality 1 of a given action is applied in a canola field. Modality 1 for actions CC, CM and W corresponds respectively to the choice of the resistant cultivar, the application of a canola management plan unfavorable to blackleg, and plowing.

We considered a regular grid of 10 by 10 fields. The first year, the land use is as follows: the top left field is in canola, and from left to right and top to bottom we repeat the same pattern, canola then wheat then barley. Then crop rotation applies yearly as described above. In this configuration, a canola field always has 2 wheat neighbors and 2 barley neighbors. The first year, all wheat fields have a low inoculum production level and a low percentage of virulent spores. For each of the 4 policies tested, we simulated 100 trajectories of length 100 of the GMDP model. Average proportions of use of each action and average proportions of state of wheat fields are plotted on Fig 5.

Code and results are available from FigShare (https://dx.doi.org/10.6084/m9.figshare.3759465.v1).

**Fig 6:**
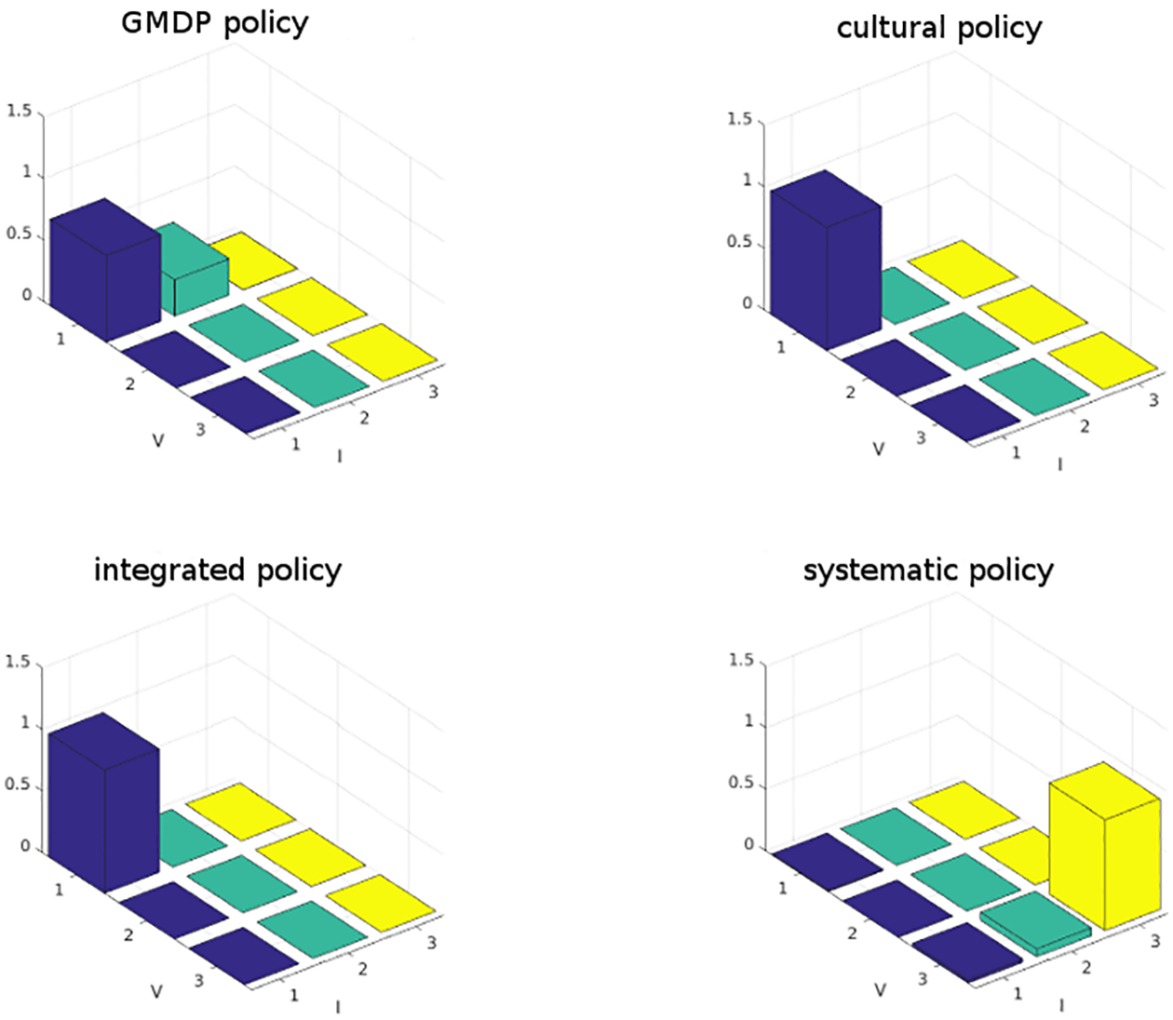
Distribution of wheat field states, in average over 100 simulations of 100 years of blackleg dynamics. Axis I indicates the level of inoculum production in the field (1 = low, 2 = medium, 3 = high), and axis V indicates the percentage of virulent spores in the inoculum (1 = low, 2 = medium, 3 = high).

We observed that even though the *integrated* policy prescribed the use of resistant cultivar for certain states of the neighborhood, these states were never reached in the simulations and the *integrated* policy was able to maintain low levels of both inoculum and virulent spores proportion. So in practice, the *integrated*, *cultural control* and GMDP policies succeeded in avoiding the development of blackleg without using the resistant cultivar while, as expected, under the *systematic* policy, the resistance was broken down and the inoculum level reached state 3 (see Fig 6). With the GMDP policy, the state medium level of inoculum and low percentage of virulent spores (*I* = 2, *V* = 1) was sometimes reached, which is not the case with the *integrated* and the *cultural control* policies. But the mean value of reward per field and per year of the GMDP policy (2503 €) was 10% larger than that of the *integrated* (2259 €) and *cultural control* (2256 €) policies and 44% larger than that of the *systematic* policy (1735 €). These results depend on the underlying assumptions of our model (in particular, that the dynamics of the disease are perfectly described by the GMDP transition model). Also, it is assumed that: i) there is a perfect knowledge of the status of each single field; ii) there is a regional coordinated decision-making; iii) fields are evenly sized and evenly spaced apart; iv) weather conditions are always conducive to sporulation; v) there is no cost to access information. These assumptions are obviously strong, but they allow to simply model the behaviour of epidemics at a regional scale. Further modeling developments could be made ni order to address these shortcomings. They also depend on the parameters values used for the simulations (described in Table 6). An analysis of the sensitivity of the GMDP policy and the contrasted ones to the model parameters values could point out situations where the use of the resistant cultivar is useful.

## Conclusion

In this article, we have presented the first toolbox dedicated to the Graph-Based Markov Decision Process (GMDP) framework. GMDPtoolbox provides a Matlab structure to encode GMDP problems, as well as modeling tools, solution algorithms, and analysis tools for evaluating and comparing policies (arbitrary policies or obtained by the provided GMDP solution algorithms). In addition, GMDPtoolbox provides a didactic toy example and an illustrative example describing a problem of sustainable collective management of plant disease at the landscape scale. This toolbox completes the set of available toolboxes for solving factored MDP. SPUDD [37] and APRICODD [38] are JAVA softwares implementing respectively exact and approximate solution approaches to FMDP, based on Algebraic Decision Diagrams. The aGrUM C++ library [39] also implements solution algorithms for factored MDP. A limit of these toolboxes is that they use frameworks and algorithms that can handle only a flat representation of the action space, while in GMDPtoolbox, the action space is multidimensional. In the blackleg of canola problem, it would mean to choose a global action among 8^100^ instead of choosing 100 actions each among 8 possible ones with the GMDP model. This is clearly not tractable. The F^3^MDP Matlab solver [19] removes this limitation, and can model any FA-MDP not just GMDP. However, the computational time for resolution is in general longer than with GMDPtoolbox.

GMDPtoolbox can be useful for two different types of users. Researchers in Artificial Intelligence can test newly developed algorithms for sequential decision in spatial context and compare them with the algorithms provided in the toolbox, on the included examples (using, for example, the toolbox analysis functions). They can also use the toolbox for teaching purposes.

Modelers can use it to support experts thinking, in many applied fields (including ecology and agriculture). The process of modeling an applied management problem in the GMDP framework and building transition and reward functions is already useful by itself to better understand the considered problems. This is something that we observed with specialists of forestry management [25], plant disease control [23] or ecology [26]. Then, the analysis of the solutions (policies) obtained by applying GMDP solution algorithms gives a further insight on the studied problem and sometimes generates new ways of managing spatio-temporal processes, or confirm the quality of the proposed expert policies.

Several extensions of GMDPtoolbox can be explored. The first one is to include the GMDP solution algorithm proposed by [21]. It is based on approximate Value Iteration and approximates the value function using a *Belief Propagation* algorithm. Another natural extension would be to develop an R version of the toolbox. The R language, initially developed for statistical analysis, is a GNU language which is now widely used by modelers, to perform computation and data analysis. An R version of GMDPtoolbox would allow to target a larger community of modeler scientists.

To conclude, we have illustrated the usefulness of GMDPtoolbox for modeling and solving problems of management in agroecology. The scope of applications goes beyond this field since the interactions do not need to be spatial interactions. The framework is also adapted for example to networks of social relationships like in viral marketing applications [21] or to computer networks like in computer virus control.

## Supporting information

S1 Appendix Transition probability functions for the GMDP model of management of blackleg of canola. (PDF)
