# Supplementary material for: GMDPtoolbox: A Matlab library for designing spatial management policies. Application to the long-term collective management of an airborne disease
Source: PLoS One. 2017 Oct 5;12(10):e0186014. doi: 10.1371/journal.pone.0186014 (PMC5628918; doi:10.1371/journal.pone.0186014)
Supplement: S1 Appendix — (PDF) [file pone.0186014.s001.pdf]

Supporting Information

Transition probability functions for the GMDP model of management of blackleg of canola

The transition probability function $P(s_i^{t+1} \mid s_{N_i}^t, a_i^t)$ in a field i sown with canola at time t and sown with wheat at time $t+1$ could be written as follow.

$$\begin{aligned} & P(s_i^{t+1} \mid s_{N_i}^t, a_i^t) \\ & = P(V_i^{t+1}, I_i^{t+1} \mid V_{NW_i}^t, I_{NW_i}^t, CC_i^t, W_i^t, CM_i^t) \\ & = P(V_i^{t+1} \mid V_{NW_i}^t, I_{NW_i}^t, CC_i^t) \times P(I_i^{t+1} \mid V_{NW_i}^t, I_{NW_i}^t, CC_i^t, W_i^t, CM_i^t) \end{aligned}$$

where NW_i is the set of index of the neighboring fields of i sown in wheat when i is in canola.

- $P(V_i^{t+1} \mid V_{NW_i}^t, I_{NW_i}^t, CC_i^t)$ **term**
  The virulence level V_i^{t+1} at time $t+1$ in the inoculum produced in a wheat field i is modeled as a deterministic function which depends only on the cultivar choice CC_i^t made in the field at time t (when the crop was canola). If $CC_i^t = 1$ (resistant cultivar), then $V_i^{t+1} = 3$, meaning $P(V_i^{t+1} = 3 \mid V_{NW_i}^t, I_{NW_i}^t, CC_i^t = R) = 1$. If $CC_i^t = 2$ (susceptible cultivar), V_i^{t+1} is set equal to a weighted mean of the levels of inoculum in the neighboring fields. The three possible states for variable I_i are associated to three classes of percentage of cankered cross-sections at the crown level: $I_i = 1$ (resp. 2, and 3) corresponds to a percentage between 0 and 25 % (resp. between 25 and 50 %, and between 50 and 100 %). We denote by $\mathcal{I}_i^t$ the median percentage of the level of inoculum at time t in field i. Similarly, the three possible states for variable V_i are interpreted as follows: $V_i = 1$ (resp. 2, and 3) corresponds to a percentage of virulent spores in the inoculum between 0 and 5 % (resp. between 6 and 50 %, and between 51 and 100 %). We denote by $\mathcal{V}_i^t$ the median percentage of the level of inoculum at time t in field i. V_i^{t+1} is then defined as the virulence level whose associated interval contains $\mathcal{V}_i^{t+1}$ with

$$\mathcal{V}_i^{t+1} = \frac{1}{\sum_{j \in NW_i^t} \mathcal{I}_j^t} \sum_{j \in NW_i^t} \mathcal{I}_j^t \, \mathcal{V}_j^t.$$

- $P(I_i^{t+1} \mid V_{NW_i}^t, I_{NW_i}^t, CC_i^t, W_i^t, CM_i^t)$ **term**
  If $W_i^t = 1$, we assume that whatever the choices for cultivar and canola management plan, plowing is efficient and decreases the inoculum level to state 1:

$$P(I_i^{t+1} = 1 \mid V_{NW_i}^t, I_{NW_i}^t, CC_i^t, W_i^t = 1, CM_i^t) = 1.$$

  If $W_i^t = 2$, then the values of the two other actions matter.

  - Case $W_i^t = 2, CC_i^t = 2$ and $CM_i^t = 2$.
    This is the combination of actions the most favorable to blackleg development. In this case $V_{NW_i}^t$ does not influence the transition since the cultivar is susceptible to both types of spores (virulent and avirulent). We modeled the transition probability $P(I_i^{t+1} \mid I_{NW_i}^t, 2, 2, 2)$ by an Ordinal Logistic Regression (OLR) model (extension of a multinomial logistic regression model to ordinal output variables [1]). In the OLR model, the explanatory variables are summaries of the information present in $I_{NW_i}^t$, namely $nI1^t$, $nI2^t$ and $nI3^t$ the numbers of wheat neighboring fields with an inoculum production level respectively in states 1, 2 and 3. The most

probable value of I_i^{t+1} for each possible value for the triple $(nI1^t, nI2^t, nI3^t)$ has been assessed by expert knowledge (see Table 1). These "observations" have been used to fit the OLR model (with the Matlab function *mnrfit*). The mode of the OLR model was in perfect adequacy with the expert mode.

- Case $W_i^t = 2, CC_i^t = 2$ and $CM_i^t = 1$.
  We assumed that canola management plan 1 will decrease the inoculum level at the end of the cropping season. We used the following transformation

$$
\begin{aligned}
&P(I_i^{t+1} = 1 \mid V_{NW_i}^t, I_{NW_i}^t, CC_i^t = 2, W_i^t = 2, CM_i^t = 1) = \\
&\qquad \sum_{l=1}^{2} P(I_i^{t+1} = l \mid V_{NW_i}^t, I_{NW_i}^t, CC_i^t = 2, W_i^t = 2, CM_i^t = 2), \\
&P(I_i^{t+1} = 2 \mid V_{NW_i}^t, I_{NW_i}^t, CC_i^t = 2, W_i^t = 2, CM_i^t = 1) = \\
&\qquad P(I_i^{t+1} = 3 \mid V_{NW_i}^t, I_{NW_i}^t, CC_i^t = 2, W_i^t = 2, CM_i^t = 2), \\
&P(I_i^{t+1} = 3 \mid V_{NW_i}^t, I_{NW_i}^t, CC_i^t = 2, W_i^t = 2, CM_i^t = 1) = 0.
\end{aligned}
$$

- Case $W_i^t = 2, CC_i^t = 1$ and $CM_i^t = 2$.
  As for the case $W_i^t = 2, CC_i^t = 2$ and $CM_i^t = 2$, we used an OLR. Here the explanatory variables are the $nIVk^t$ for $k = 1$ to 9, with $nIV1^t$ the number of wheat neighboring fields with state $(I_j^t, V_j^t) = (1, 1)$, $nIV2^t$ the number of wheat neighboring fields with state $(I_j^t, V_j^t) = (1, 2)$, and so on until $nIV9^t$ the number of wheat neighboring fields with state$(I_j^t, V_j^t) = (3, 3)$. Several combinations of values have been submitted to an expert who assessed the corresponding most probable state for I_i^{t+1} (see Table 2). The mode of the OLR model was in adequacy with the expert mode for 119 cases among the 132 (90%).
- Case $W_i^t = 2, CC_i^t = 1$ and $CM_i^t = 1$.
  Again, we assumed that canola management plan 1 will decrease the inoculum production level at the end of the cropping season.

$$
\begin{aligned}
&P(I_i^{t+1} = 1 \mid V_{NW_i}^t, I_{NW_i}^t, CC_i^t = 1, W_i^t = 2, CM_i^t = 1) = \\
&\qquad \sum_{l=1}^{2} P(I_i^{t+1} = l \mid V_{NW_i}^t, I_{NW_i}^t, CC_i^t = 1, W_i^t = 2, CM_i^t = 2), \\
&P(I_i^{t+1} = 2 \mid V_{NW_i}^t, I_{NW_i}^t, CC_i^t = 1, W_i^t = 2, CM_i^t = 1) = \\
&\qquad P(I_i^{t+1} = 3 \mid V_{NW_i}^t, I_{NW_i}^t, CC_i^t = 1, W_i^t = 2, CM_i^t = 2), \\
&P(I_i^{t+1} = 3 \mid V_{NW_i}^t, I_{NW_i}^t, CC_i^t = 1, W_i^t = 2, CM_i^t = 1) = 0.
\end{aligned}
$$

$nI1^t$	$nI2^t$	$nI3^t$	I_i^{t+1} mode
0	0	1	3
0	0	2	3
0	0	3	3
0	0	4	3
0	1	0	2
0	1	1	3
0	1	2	3
0	1	3	3
0	2	0	2
0	2	1	3
0	2	2	3
0	3	0	3
0	3	1	3
0	4	0	3
1	0	0	1
1	0	1	3
1	0	2	3
1	0	3	3
1	1	0	2
1	1	1	3
1	1	2	3
1	2	0	3
1	2	1	3
1	3	0	3
2	0	0	1
2	0	1	3
2	0	2	3
2	1	0	2
2	1	1	3
2	2	0	3
3	0	0	2
3	0	1	3
3	1	0	2
4	0	0	2

Table 1. Expert information used to fit the ordinal logistic regression model of the probability of I_i^{t+1} conditionally to $I_{NW_i}^t$ when $W_i^t = 2$, $CC_i^t = 2$, and $CM_i^t = 2$.
Explanatory variables are $nI1^t$, $nI2^t$ and $nI3^t$ the number of wheat neighboring fields with an inoculum production level respectively in state 1, 2 and 3.

$nIV1^t$	$nIV2^t$	$nIV3^t$ 1	$nIV4^t$	$nIV5^t$	$nIV6^t$	$nIV7^t$	$nIV8^t$	$nIV9^t$	I_i^{t+1} mode
0	0	0	0	0	0	0	0	1	3
0	0	0	0	0	0	0	1	0	3
0	0	0	0	0	0	1	0	0	1
0	0	0	0	0	0	0	0	2	3
0	0	0	0	0	0	0	1	1	3
0	0	0	0	0	0	0	2	0	3
0	0	0	0	0	0	1	0	1	3
0	0	0	0	0	0	1	1	0	3
0	0	0	0	0	0	2	0	0	2
0	0	0	0	0	0	0	0	3	3
0	0	0	0	0	0	0	1	2	3
0	0	0	0	0	0	0	2	1	3
0	0	0	0	0	0	0	3	0	3
0	0	0	0	0	0	1	0	2	3
0	0	0	0	0	0	1	1	1	3
0	0	0	0	0	0	1	2	0	3
0	0	0	0	0	0	2	0	1	3
0	0	0	0	0	0	2	1	0	3
0	0	0	0	0	0	3	0	0	2
0	0	0	0	0	0	0	0	4	3
0	0	0	0	0	0	0	1	3	3
0	0	0	0	0	0	0	2	2	3
0	0	0	0	0	0	0	3	1	3
0	0	0	0	0	0	0	4	0	3
0	0	0	0	0	0	1	0	3	3
0	0	0	0	0	0	1	1	2	3
0	0	0	0	0	0	1	2	1	3
0	0	0	0	0	0	1	3	0	3
0	0	0	0	0	0	2	0	2	3

Continued on next page

Following of previous page

$nIV1^t$	$nIV2^t$	$nIV3^t$ 1	$nIV4^t$	$nIV5^t$	$nIV6^t$	$nIV7^t$	$nIV8^t$	$nIV9^t$	I_i^{t+1} mode
0	0	0	0	0	0	2	1	1	3
0	0	0	0	0	0	2	2	0	3
0	0	0	0	0	0	3	0	1	3
0	0	0	0	0	0	3	1	0	3
0	0	0	0	0	0	4	0	0	2
0	0	0	0	0	1	0	0	0	3
0	0	0	0	1	0	0	0	0	2
0	0	0	1	0	0	0	0	0	1
0	0	0	0	0	1	0	0	1	3
0	0	0	0	1	0	0	0	1	3
0	0	0	0	0	1	0	1	0	3
0	0	0	0	1	0	0	1	0	3
0	0	0	1	0	0	0	0	1	3
0	0	0	0	0	1	1	0	0	3
0	0	0	1	0	0	0	1	0	2
0	0	0	0	1	0	1	0	0	2
0	0	0	1	0	0	1	0	0	1
0	0	0	0	1	0	0	2	0	3
0	0	0	1	0	0	0	0	2	3
0	0	0	0	0	1	1	0	1	3
0	0	0	1	0	0	0	1	1	3
0	0	0	0	0	1	1	1	0	3
0	0	0	0	1	0	1	0	1	3
0	0	0	1	0	0	0	2	0	3
0	0	0	0	1	0	1	1	0	3
0	0	0	1	0	0	1	0	1	3
0	0	0	0	0	1	2	0	0	3
0	0	0	1	0	0	1	1	0	3
0	0	0	0	1	0	2	0	0	3
0	0	0	1	0	0	2	0	0	2
0	0	0	0	0	1	0	0	3	3
0	0	0	0	1	0	0	0	3	3
0	0	0	0	0	1	0	1	2	3
0	0	0	0	1	0	0	1	2	3
0	0	0	0	0	1	0	2	1	3
0	0	0	0	1	0	0	2	1	3
0	0	0	0	0	1	0	3	0	3
0	0	0	1	0	0	0	0	3	3
0	0	0	0	0	1	1	0	2	3
0	0	0	1	0	0	0	1	2	3
0	0	0	0	1	0	1	0	2	3
0	0	0	0	0	1	1	1	1	3
0	0	0	1	0	0	0	2	1	3
0	0	0	0	1	0	1	1	1	3
0	0	0	0	0	1	1	2	0	3
0	0	0	0	0	2	0	0	0	3
0	0	0	0	1	1	0	0	0	3
0	0	0	0	2	0	0	0	0	3
0	0	0	1	0	1	0	0	0	3
0	0	0	2	0	0	0	0	0	2
0	0	1	0	0	0	0	0	0	2
0	1	0	0	0	0	0	0	0	1
1	0	0	0	0	0	0	0	0	1
0	0	1	0	0	0	0	0	1	3
0	1	0	0	0	0	0	0	1	3
0	0	1	0	1	0	0	0	0	3
0	1	0	0	0	0	0	1	0	2
1	0	0	0	0	0	0	0	1	2
0	0	1	0	0	0	1	0	0	2
1	0	0	0	0	0	0	1	0	3
0	1	0	0	0	0	1	0	0	2
1	0	0	0	0	0	1	0	0	1
0	0	1	0	0	1	0	0	0	3
0	1	0	0	0	1	0	0	0	3
0	0	1	0	1	0	0	0	0	3
0	1	0	0	1	0	0	0	0	3
1	0	0	0	0	1	0	0	0	3
0	0	1	1	0	0	0	0	0	2
1	0	0	0	1	0	0	0	0	2
0	1	0	1	0	0	0	0	0	1
1	0	0	1	0	0	0	0	0	1
0	0	2	0	0	0	0	0	0	2
0	1	1	0	0	0	0	0	0	2
0	2	0	0	0	0	0	0	0	1

Continued on next page

Following of previous page

$nIV1^t$	$nIV2^t$	$nIV3^t$	$nIV4^t$	$nIV5^t$	$nIV6^t$	$nIV7^t$	$nIV8^t$	$nIV9^t$	I_i^{t+1} mode
1	0	1	0	0	0	0	0	0	2
1	1	0	0	0	0	0	0	0	1
2	0	0	0	0	0	0	0	0	1
0	0	2	0	0	0	0	0	1	3
2	0	0	0	0	0	1	0	0	2
0	0	3	0	0	0	0	0	0	3
3	0	0	0	0	0	0	0	0	1
0	0	4	0	0	0	0	0	0	3
4	0	0	0	0	0	0	0	0	1
0	0	0	1	0	0	0	3	0	3
0	0	0	0	1	0	1	2	0	3
0	0	0	1	0	0	1	0	2	3
0	0	0	0	0	1	2	0	1	3
0	0	0	1	0	0	1	1	1	3
0	0	0	0	1	0	2	0	1	3
0	0	0	0	1	0	2	1	0	3
0	0	0	1	0	0	1	2	0	3
0	0	0	0	1	0	2	1	0	3
0	0	0	1	0	0	2	0	1	3
0	0	0	0	0	1	3	0	0	3
0	0	0	1	0	0	2	1	0	3
0	0	0	0	1	0	3	0	0	3
0	0	0	1	0	0	3	0	0	3
0	0	0	0	0	1	0	0	2	3
0	0	0	0	1	0	0	0	2	3
0	0	0	0	0	1	0	1	1	3
0	0	0	0	1	0	0	1	1	3
0	0	0	0	0	1	0	2	0	3

Table 2. Expert values used to fit the ordinal logistic regression model of the probability of I_i^{t+1} conditionally to $I_{NW_i}^t$ when $W_i^t = 2$, $CC_i^t = 1$, and $CM_i^t = 2$.
Explanatory variables are $nIV1^t$ to $nIV9^t$, which are the number of wheat neighboring fields in state 1 to 9.

References

1. Agresti A. Categorial Data Analysis. John Whiley and Son; 2002.
